# Recent progress in stimuli‐activable metallo‐prodrugs for cancer therapy

**DOI:** 10.1002/smo.20240030

**Published:** 2024-08-29

**Authors:** Jinzhe Liang, Fangmian Wei, Hui Chao

**Affiliations:** ^1^ MOE Key Laboratory of Bioinorganic and Synthetic Chemistry Guangdong Basic Research Center of Excellence for Functional Molecular Engineering School of Chemistry Sun Yat‐Sen University Guangzhou China; ^2^ State Key Laboratory for the Chemistry and Molecular Engineering of Medicinal Resources MOE Key Laboratory for Chemistry and Molecular Engineering of Medicinal Resources Collaborative Innovation Center for Guangxi Ethnic Medicine School of Chemistry and Pharmaceutical Sciences Guangxi Normal University Guilin China; ^3^ MOE Key Laboratory of Theoretical Organic Chemistry and Functional Molecule School of Chemistry and Chemical Engineering Hunan University of Science and Technology Xiangtan China

**Keywords:** cancer therapy, metallo‐prodrugs, stimuli‐activable

## Abstract

The clinical approval of platinum‐based drugs has prompted the development of novel metallo‐complexes during the last several decades, while severe problems, especially for poor water solubility, drug resistance and toxicity in patients, greatly hindered the clinical trials and curative efficacy. To address these issues, the concept of metallo‐prodrugs has been proposed for oncology. Some stimuli‐activable metallo‐prodrugs provide new insights for designing and preparing site‐specific prodrugs with maximized therapeutic efficacy and negligible unfavorable by‐effects. In this review, recent progress in stimuli‐activable metallo‐prodrugs in the past 20 years has been overviewed, where endogenous and exogenous stimuli have been involved. Typical examples of smart stimuli‐activable metallo‐prodrugs are discussed regarding to their molecular structure, activation mechanism, and promising biomedical applications. In the end, challenges and future perspectives in metallo‐prodrugs have been discussed.

## Graphical abstract

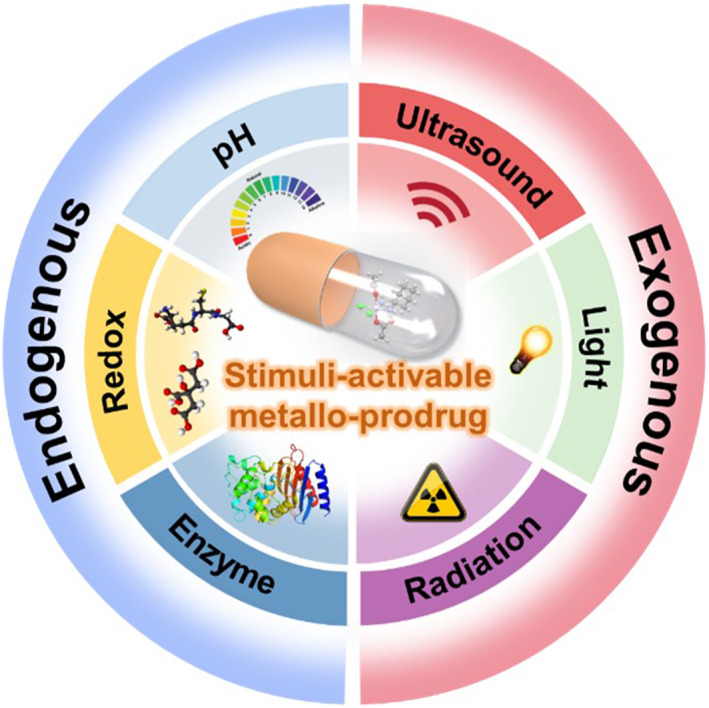

## 1 INTRODUCTION

Metal ions are essential in a wide spectrum of biochemical processes, such as metabolism, enzymatic catalysis, and osmotic regulation.^[1]^ The historical records of metal‐based drugs can be found dating back to the four oldest civilizations, Mesopotamia, Indus Valley, Ancient Egypt and China. At that ancient period, people had realized the values of gold, silver, and copper for therapeutic proposals. Notably, the era of metallo‐drugs started in the 1970s with the United States Food and Drug Administration (FDA) approval of cisplatin, the first‐generation of platinum anti‐cancer drug.^[2]^ Later, a wide range of metal‐based complexes were explored as potential metallo‐drugs in diagnosis and therapy against different diseases, including inflammation, diabetes, cancer, and cardiovascular disease, etc.^[3]^

Nowadays, even though small molecules are a mainstay of the pharmaceutical market, metallo‐drugs have garnered great attention owing to their fascinating and distinct characteristics compared to those of purely organic drugs. The rational design of bioactive metallodrugs can endow them with unique chemical and biological diversities, which are infeasible for small‐molecule drugs.^[4]^ Firstly, depending on the coordination numbers of the central metal atoms, the metal‐based complexes possess different geometries, such as octahedral, trigonal bipyramidal, tetrahedral, and square planar shapes, offering a larger stereochemical diversity than the carbon atom with a simple tetrahedral shape.^[3a]^ This diversity can facilitate the biological target issue and confer a better drug selectivity.^[5]^ Secondly, the multivalent metal ions make them undergo the redox reactions and regulate the biological processes under the reducing environment.^[6]^ Thirdly, metal complexes with luminescent central metals are endowed with superior optical properties, thereby exerting great diagnostic potential.^[7]^ Particularly, metallo‐drugs composed with magnetic and radioactive metals have been widely utilized as contrast agents in spectroscopic diagnosis. Nevertheless, compared with the organic counterparts, the widespread use and clinical utilization of metal complexes encounter severe problems, including poor water solubility, unfavorable biodistribution, drug resistance, and toxicity in patients.^[8]^

**SCHEME 1:**
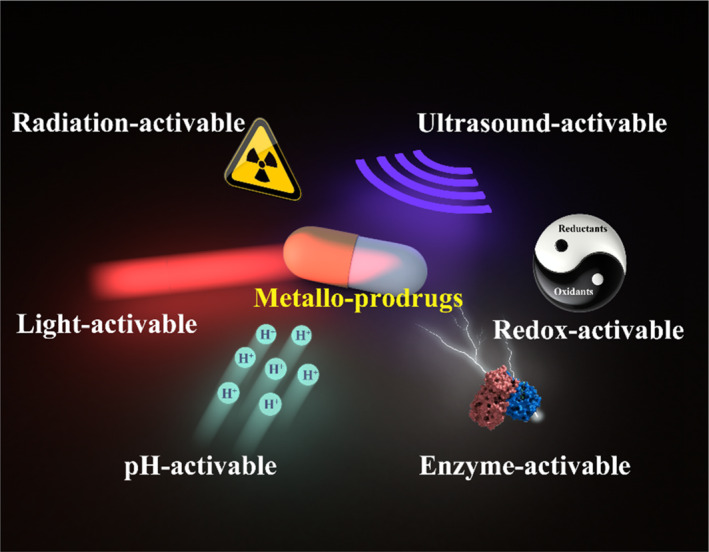
Schematic illustration of stimuli‐responsive metallo‐prodrugs for cancer therapy.

In 1958, the term “prodrug” was first introduced by Adrien Albert to prepare chemically modified inactive compounds derived from drugs that can undergo in vivo biotransformation with the release of the pharmacologically active drug form.^[9]^ Nowadays, the ideal prodrug can achieve controlled release of parent drug along with the ligand removal once administered in a living organism, thereby achieving target‐specific delivery, enhancing the biocompatibility, physiochemical, and pharmacokinetic characteristics.^[10]^ Appropriate triggers are of great significance for the activation of prodrugs. Typically, the triggers for ligand cleavage and accurate delivery of bioactive drugs to the tumor tissues can be divided into two major categories: endogenous and exogenous stimuli. Specifically, the endogenous stimuli (e.g., weakly acidic pH, overexpressed enzymes, redox gradient, and hypoxic tumor microenvironment) are strongly associated with the pathological property, while the exogenous stimuli (e.g., light, ultrasound, and X‐ray radiation) are employed for remote regulation of prodrug activation (Scheme 1). In the 2000s, the concept of “metallo‐prodrug” was proposed, which was mainly developed for the potential applications in oncology.^[11]^ The stimuli‐activable metallo‐prodrug combines the edges of the metallo‐drug and prodrug, possessing the manifold molecular structures and exhibiting potential for more effective administration and by‐effect reduction in future clinical trials.

**FIGURE 1:**
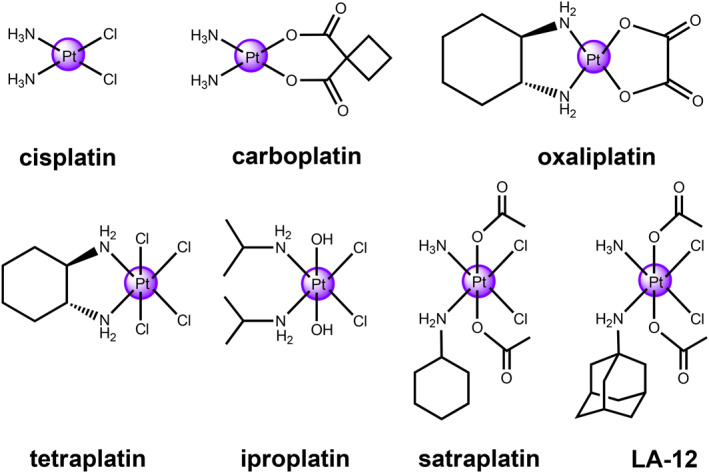
FDA‐approved anti‐cancer Pt(II) metallodrugs and Pt(IV) complexes in clinical trials.

Anti‐cancer cisplatin is one of the most commonly utilized chemotherapeutic metallodrugs in first‐line clinical use. In addition to cisplatin, its analogs carboplatin and oxaliplatin are also a part of FDA‐approved platinum‐based chemotherapeutic agents against many types of cancers, including the lung, breast, colorectal, ovarian and testicular cancers (Figure 1).^[1a]^ Their corresponding action includes entering across cellular membrane, generating platinated DNA cross‐linkers, thereby triggering apoptotic cell death.^[12]^ Nevertheless, the curative efficacy of Pt(II)‐based chemotherapy are greatly hindered by their strong cytotoxicity, intrinsic drug resistance, and adverse by‐effects originated from the aquated resultant and non‐selective covalent binding with bioactive molecules.^[13]^ To address these severe issues, there is growing attention in exploring stimuli‐responsive Pt(IV) prodrugs with a six‐coordinated octahedral configuration, which can decrease the unfavorable reactions and prevent the off‐target interactions before DNA binding.^[14]^ The design of the Pt(IV) prodrugs is mainly building upon cytotoxic Pt(II) structures, especially for cisplatin and other FDA‐approved Pt(II) species. Abundant Pt(IV) complexes have been developed as potential chemotherapeutic metallodrugs in the past 20 years. Particularly, tetraplatin, iproplatin, satraplatin, and LA‐12 have been investigated in the clinical trials (Figure 1). All of the above‐mentioned anti‐cancer Pt(IV) agents are derivatives of cisplatin which maintains the integrity of [*cis*‐Pt(NH_3_)_2_Cl_2_] with a similar coordination sphere. Despite that none of them have been applied in the clinical use so far, the initial investigations in this field have greatly propelled the advances of these Pt(IV) complexes for cancer therapy.

**SCHEME 2:**
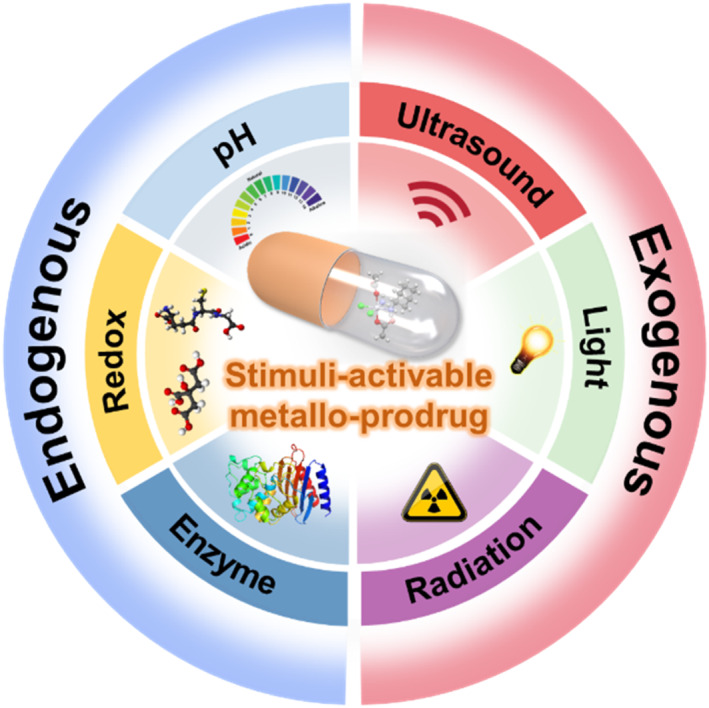
Schematic illustration of endogenous‐ and exogenous‐stimuli‐activable metallo‐prodrugs for cancer therapy.

In this review, recent cutting‐edge studies in investigating smart stimuli‐activable metallo‐prodrugs for cancer diagnosis and therapy have been discussed (Scheme 2). Based on the stimulus types, the metallo‐prodrugs are mainly divided into three categories: endogenous‐stimuli‐activated, exogenous‐stimuli activated, and dual‐/multi‐stimuli activated metallo‐prodrugs. The structure, synthetic approach into nanoscale formulations, responsive mechanism of metallo‐prodrugs and the strategies of accurately manipulating the stimuli are discussed in each subsection. Finally, challenges along with the rapid advances of stimuli‐responsive metallo‐prodrugs and the future perspectives are provided in the last section. We hope this review can offer useful guidance for researchers in this field to further promote the progress on smart stimuli‐responsive metallo‐prodrugs.

## 2 ENDOGENOUS‐STIMULI‐ACTIVATED METALLO‐PRODRUGS

Tumor tissues are composed of transformed and non‐transformed tumor cells, cooperating by varied mechanisms to keep the tumor microenvironment suitable for the progression and metastasis of malignant cancer cells. Consequently, the microenvironment of tumor tissues is largely different from that of normal tissues from many aspects, especially for the distinctive pathophysiological features, such as relatively low pH value,^[15]^ overexpression of specific enzymes,^[16]^ hypoxia,^[17]^ cellular oxidants,^[18]^ and reduced condition owing to the cellular reductants, that is, elevated level of glutathione (GSH) and ascorbic acid. These intracellular features could be employed as endogenous stimuli for metallo‐prodrugs to evoke their pharmacological effect but not being detrimental to the normal tissues.^[19]^

To sum up, the above abnormalities of tumor microenvironment can act as stimuli for rational design and construction of smart stimuli‐activable metallo‐prodrugs. A series of endogenous‐stimuli‐activated metallo‐prodrugs triggered by low pH, specific enzymes, and redox reactions have been explored through this strategy. Herein, an overview of smart metallo‐prodrugs is provided building upon the distinctive pathophysiological markers in the tumor microenvironment in this section.

### 2.1 pH

The most unique feature of tumor microenvironment is the acidic extracellular interstitial fluids caused by glycolysis under hypoxia, which plays a crucial part in the occurrence, progression, and drug resistance issue at tumor tissues.^[3a]^ The pH value at extracellular normal cell is approximately 7.4, while that at the extracellular cancer cell is approximately 6.9, which is a universal trait of in any types of solid tumors at different stages. The physiological intracellular pH in cancer cells (pH_i_ = 7.5) is a little bit higher than that in normal cells (pH_i_ = 7.2), while the pH value at lysosomes in cancer cells (pH = 4.5–5.5) is lower than that in normal counterparts (pH = 5.0–6.0), providing a slightly acidic condition in cancer cell cytoplasm. The abnormal pH gradient in intracellular and extracellular tumor tissues widely exists in diverse solid tumor types. Hence, they can probably act as stimuli for pH‐responsive metallo‐prodrugs owing to the acidic tumor extracellular matrix.

**FIGURE 2:**
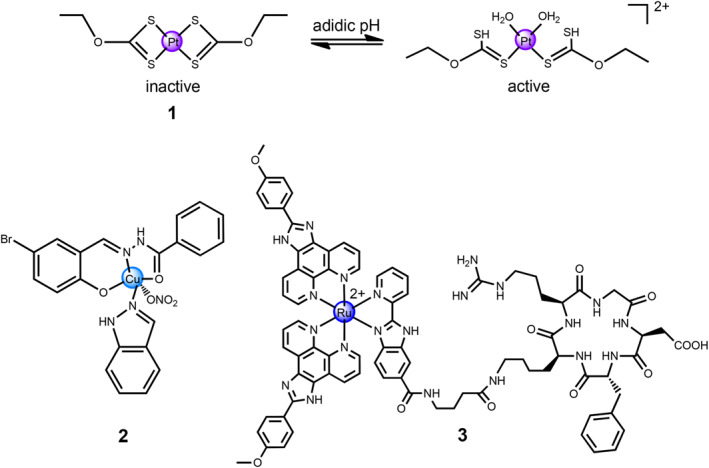
Chemical structures of pH‐responsive metallo‐prodrugs **1**–**3**.

An early paradigm of such pH‐responsive anti‐cancer platinum‐based metallo‐prodrug was the conjugation of *O*‐dithiocarbonic acid at the axial position for Pt(II) complex **1** (Figure 2).^[15a]^ Since the binding affinity of S element for Pt center metal is stronger than that of the amino groups, Pt(II) complex **1** was inactive for the DNA platination. Nevertheless, since *O*‐dithiocarbonic acid is instable and two S atoms within *O*‐dithiocarbonic acid undergo protonation under a weakly acidic environment, the breakdown of Pt‐S bonds results in the generation of an active species, aqua form of Pt(II) complex **1**. After the pH value rises, protons dissociated from the S atoms, thereby recovering the inactive species, thioplatin **1**. The pH‐activable interaction of thioplatin **1** with DNA was proved by electrophoretic mobility of the archetypal plasmid DNA. Notably, cellular uptake of Pt species was pH‐independent, while the interaction of thioplatin with DNA was around eight times higher at pH = 6.8 than at pH = 7.4. Moreover, thioplatin **1** showed an excellent in vivo anti‐tumor effect with negligible adverse effects on the major organs under slightly acidic conditions.

Human serum albumin (HSA), as the predominant transport protein existing in human intravascular space, can function as the ideal nanocarrier for drug delivery owing to its distinct advantages, for example, suitable biocompatibility, low toxicity, and diminished inflammatory stimulation.^[20]^ By changing the two potential leaving groups NO_3_^−^ and indazole, a Cu(II) complex **2** was tethered to the hydrophobic cavity at the IIA subdomain site of HSA *via* the coordination of Cu^2+^ with Lys199 and His242 (Figure 2).^[15b]^ HSA carrier binding to the Cu(II) complex **2** prefers to accumulate into the leaky tumor vasculature through enhanced permeability and retention (EPR) effect, thereby achieving the controlled delivery of Cu(II) complex **2**. Nevertheless, the coordination strength between HSA and Cu^2+^ would be significantly decreased in weakly acidic conditions, followed by the release of Cu(II) complex to damage DNA within cancer cells, causing the p53 pathway activation, G2/M phase cell cycle arrest, and mitochondrial dysfunction, thereby inducing cancer cell death by the apoptotic pathway. The HSA carrying with Cu(II) complex displayed two times larger cytotoxicity than that of Cu(II) complex alone against MCF‐7 cancerous cells and showed enhanced activity to inhibit the growth of tumor.

A successful development of a targeted drug delivery can greatly enhance the therapeutic effect of anti‐cancer metallo‐prodrugs by minimizing the adverse effects and the undesirable adsorption. These novel strategies for the design of theranostic anti‐cancer agents for the utilization in cancer theranostics have attracted substantial attention. Zhao et al. developed a luminescent Ru(II) prodrug modified with the cyclic Arg‐Gly‐Asp (RGD)‐peptide and the pH‐sensitive benzimidazole‐based ligand, termed Ru(II) complex **3** (Figure 2), resulting in the prodrug with cervical tumor‐targeting ability and improved therapeutic efficacy.^[15c]^ In the weakly acidic tumor microenvironment (pH = 6.8), the active Ru complex and the ligand were released from Ru(II) complex **3**. The Ru(II) complex **3** displayed higher cytotoxicity against malignant cancerous cell lines (CaSki, SiHa, and Hela cells) compared with that of normal cells (Ect1/E6E7 cells). After 3‐h incubation with the Ru(II) complex **3**, strong two‐photon luminescence was emitted across the 3D CaSki multicellular tumor spheroids (MCTSs), proving the good penetration ability of two‐photon luminescence. DNA fragmentation was observed in the majority of CaSki cells when treated with complex **3**, while the DNA of most Ect1/E6E7 cells remained intact. The in vivo experiments revealed that the Ru(II) complex **3** preferred to accumulate in tumor tissues rather than in other organs of CaSki tumor‐bearing nude mice, resulting in enhanced anti‐tumor effects but negligible lesions to the vital organs. Therefore, Ru(II) prodrug **3** is a potential candidate as an anti‐cancer theranostic agent.

### 2.2 Enzymes

During the disease occurrence and development processes, for example, cancer, inflammation, and neurodegenerative disorders, the concentration levels of specific enzymes are usually highly expressed under certain physiological conditions. For instance, the expression levels of many enzymes, such as esterase, protease, azoreductase, β‐lactamase, and β‐glucuronidase, in pathological tissues during illnesses are usually elevated than those in the normal counterparts. Such enzyme level gradients can be used as stimuli to design smart enzyme‐responsive metallo‐prodrug systems in cancer theranostics.^[21]^

**FIGURE 3:**
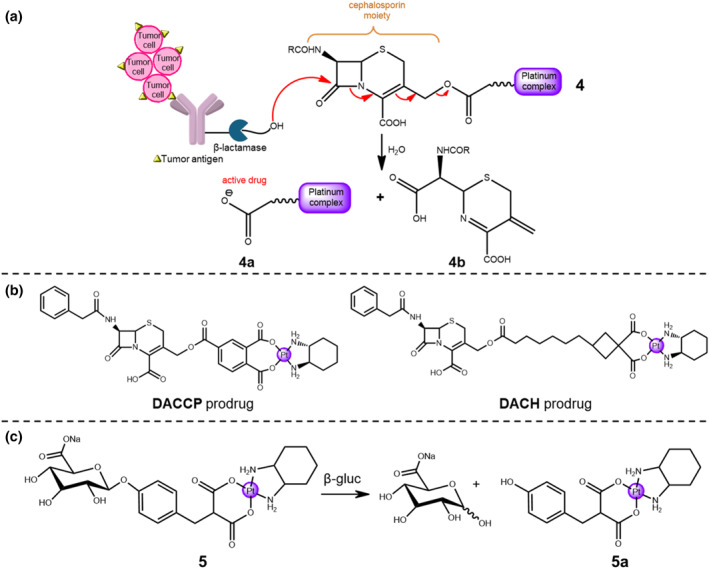
(a) Mechanism scheme of the ADEPT system utilizing β‐lactamase enzyme and platinum complex. (b) Chemical structures of two anti‐tumor Pt prodrugs, **DACCP** and **DACH**. (c) Hydrolysis reaction of a β‐glucuronide‐based Pt metallo‐prodrug **5**.

Nowadays, the platinum‐based prodrugs are widely used as chemotherapeutic agents. Nevertheless, their low water solubility and limited selectivity for cancer cells greatly hinder their curative efficacy. The pioneering work in this field is the development of β‐lactamase‐triggered metallo‐prodrug for cancer therapy. With the purpose of precisely delivering an anti‐cancer drug to the tumor tissue, Hanessian and Wang reported an ADEPT system by using β‐lactamase enzyme as the stimuli for the controlled release of platinum‐based complex in malignant tumors (Figure 3a).^[16a]^ With this aim, metallo‐prodrugs **4** conjugated with β‐lactamase to monoclonal antibodies were designed with two platinum complexes **DACCP** and **DACH**, resulting in two potential anti‐tumor candidates, the 4′‐carboxyphthalato(1,2‐cyclohexanediamine) platinum (**DACCP**) with cephalosporin subunit and the 1,2‐diaminocyclohexaneplatinum (**DACH**) complex (Figure 3b). It was expected that the enzymatic cleavage could result in the release of a carboplatin‐based prodrug linked at the 3′‐position of the cephalosporin derivative. The β‐lactamase catalyzed hydrolysis reaction of the DACCP derivative was investigated by ^1^H NMR study, proving the efficient release of two moieties, that is, the active platinum‐based drug **4a** and **4b**.

Later, using a similar strategy, Tromp et al. synthesized the hydrophilic prodrug **5** attached with β‐glucuronide moiety for the controlled delivery of active platinum complex to the malignant tumor tissues (Figure 3c).^[16b]^ Notably, β‐glucuronidase, an overexpressed enzyme in a large number of solid tumors, can be released from necrotic cancer cells.^[22]^ Therefore, selective hydrolysis of the glucuronide derivative **5** should result in the controlled release of the platinum‐based drug **5a** in the extracellular matrix of tumor tissues. Subsequently, the platinum‐based drug **5a** penetrates the cell membrane passively into the cancer cells to display its cytotoxic activity. The enzymatic hydrolysis reaction of prodrug **5** was investigated by ^1^H NMR instrument, confirming the enzymatic cleavage of β‐glucuronidase and the generation of platinum drug **5a**. This study reported the release of platinum drug under β‐glucuronidase stimulation from platinum‐based metallo‐prodrug for the first time, thereby paving the way for enhancing the selectivity and sensitivity of chemotherapy. However, there were no biological experimental results to further verify the feasibility of this enzymatic‐triggered method.

Although great developments have been achieved in enzyme‐activable metallo‐prodrugs during past decades, efforts need to be devoted for the clinical translation in the future. The next generations of enzyme‐activable metallo‐prodrugs could involve their organic counterparts, which are utilized in human beings. For example, antibody‐drug conjugates (ADCs) conjugated with an enzyme‐activable linker achieves the selective accumulation as well as the controlled drug release in the malignant cancer cells. Therefore, enzyme‐sensitive metallo‐prodrug systems can be conjugated with a targeting linker like antibody to increase their anti‐cancer activity as therapeutic drugs.

### 2.3 Redox reactions

#### 2.3.1 Cellular oxidants‐activable metallo‐prodrugs

**FIGURE 4:**
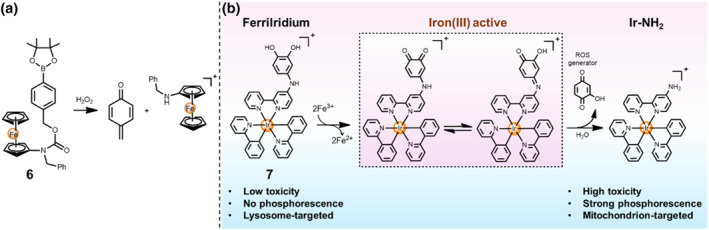
(a) H_2_O_2_‐activable aminoferrocene prodrug **6**. (b) Schematic illustration of the Fe^3+^‐responsive FerriIridium **7**.

It is worth noting that Pt(IV) prodrugs can be triggered by cellular reductants and reduced to their Pt(II) metabolites through the complete removal of two axial ligands. Distinct mechanisms of metallo‐prodrug action have received increasing attention. Iron element belongs to group VIIIb in the Periodic Table of Elements, which is the same group as ruthenium and osmium elements. The redox‐activable iron‐based complexes follow a similar mechanism of the anti‐cancer activity, which is that the cytotoxicity for DNA damage derives from the generated reactive oxygen species (ROS) and/or other toxic species instead of direct binding to DNA as platinum‐based complexes. The iron‐based complex was triggered by oxidation to form toxic quinone methide, which can occur alkylation with GSH, thereby resulting in the depletion of GSH and the destruction of the anti‐oxidative system of cancer cells.^[23]^ Following a similar method, Mokhir and co‐workers reported a H_2_O_2_‐responsive aminoferrocene‐based prodrug **6** to generate toxic *p*‐quinone methides (Figure 4a). Moreover, aminoferrocene can act as a catalyst for ROS formation, thereby accelerating the generation of •OH radicals. Thanks to the synergistic effects, the H_2_O_2_‐activable complex **6** showed high cytotoxicity against HL‐60 malignant cancerous cells (IC_50_ = 9 μM), while it displayed mild cytotoxicity against the normal fibroblast cells (IC_50_ > 100 μM).

In another study, Chao's group reported a smart Fe^3+^‐responsive catechol‐based Ir(III) metallo‐prodrug **7**, termed FerriIridium, for gastric cancer theranostics.^[18]^ In terms of the FerriIridium composition, the *meta*‐imino catechol was attached to an amino bipyridyl Ir(III) complex (Ir‐NH_2_). The catechol moiety can strongly bind to and be oxidized by free Fe^3+^ ions within the lysosome labile Fe^3+^ pool of AGS gastric cancer cells (Figure 4b). Notably, FerriIridium displayed mild cytotoxicity against LO2 and MCF‐10A normal cell lines both with IC_50_ value > 200 μM, while it showed selectively higher cytotoxicity against AGS (IC_50_ = 9.22 μM) and MKN‐28 (IC_50_ = 11.3 μM) gastric cancer lines. Activated by the free Fe^3+^ ions under acidic conditions, FerriIridium underwent an accelerated hydrolysis process into an Ir‐NH_2_ and 2‐hydroxybenzochinon in lysosomes, resulting in the generation of Fe^2+^ ions and •OH, and lysosome damage under the existence of H_2_O_2_. The as‐synthesized Ir‐NH_2_ subsequently moved to mitochondria, led to mitochondrial dysfunction and emitted strong phosphorescence, inducing cell death through apoptosis. Compared with FerriIridium, Ir‐NH_2_ demonstrated indiscriminate and high cytotoxicity (IC_50_ = 1.2–4.8 μM) against varied cell lines. Moreover, FerriIridium was more effective than 5‐Fluorouracil and showed low adverse effects towards major organs in the AGS xenograft mice model.

#### 2.3.2 Cellular reductants‐activable metallo‐prodrugs

It is worth noting that Pt(IV) prodrugs can be triggered by cellular reductants and reduced to their Pt(II) metabolites through the complete removal of two axial ligands *via* inner‐ and outer‐sphere electron transfer mechanism.^[3a]^ Therefore, the redox potentials are one of the key factors influencing the efficiency of redox‐responsive Pt(IV) prodrugs, which are matchy with that of biologically active cellular reductants, for example, GSH and ascorbic acid. It is an efficient strategy to regulate the two ligands at the axial position for modulating the redox potential, which can simultaneously modify the pharmacological features of Pt(IV) complexes. In general, the redox‐activable Pt(IV) prodrug can be allocated into three categories based on the characteristics of ligands: (1) complexes with the axial ligands possessing the targeting ability; (2) complexes with biologically active moieties; (3) complexes with biologically inactive moieties, for example, chlorides, hydroxides, and acetates.

**FIGURE 5:**
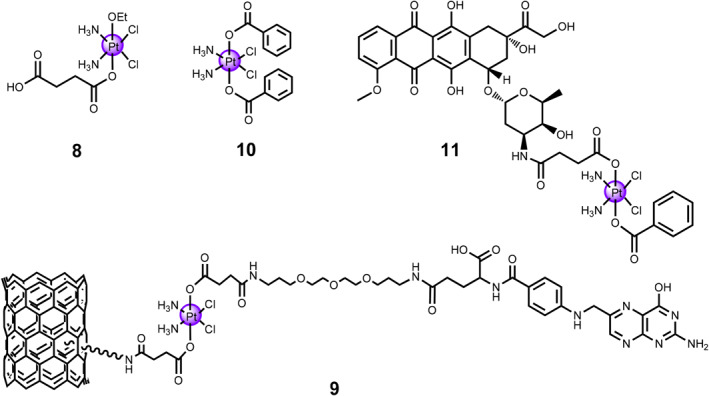
Chemical structures of redox‐activable Pt(IV) prodrugs **8–11** conjugated with SWCNT/MWCNT.

In the past few decades, single‐/multi‐walled carbon nanotubes (MWCNTs) have been explored as carriers in drug delivery systems for Pt(IV) metallo‐prodrugs **8–11** (Figure 5). In 2007, the pioneering study was reported by Lippard's and Dai's groups.^[24]^ Pt(IV) complex **8** was conjugated to the surface of single‐walled carbon nanotube (SWCNT). The SWCNT‐Pt(IV) resultants displayed great enhancement in cytotoxicity compared to Pt(IV) complex **8** alone. Notably, the cytotoxicity of the SWCNT‐Pt(IV) conjugate was larger than that of cisplatin alone. This study elucidated that SWCNTs can function as drug carriers to deliver Pt(IV) complexes into the cellular membranes, subsequently releasing active Pt(II) drug under the reduced environment. Building upon this investigation, they further prepared targeted SWCNT‐mediated Pt(IV) prodrug **9** linked with the folate derivative at axial position.^[25]^ Notably, folic acid (FA) is capable of targeting the tumor cells with high expression of folate receptor (FR). Using a similar strategy, complex **9** was conjugated to SWCNT *via* many amide linkages as that of complex **8**. The as‐obtained complex exerted efficient selective cytotoxicity against FR(+) cells. The SWCNTs function as the delivery vehicles, delivering the FA‐bearing Pt(IV) **9** into tumor cells by endocytosis and releasing active Pt(II) drugs when Pt(IV) prodrugs were exposed upon cellular reductants. Notably, it has been observed that the 1,2‐intrastrand d(GpG) cross‐linked between the released cisplatin and nuclear DNA.

In addition to SWCNT, the MWCNT can function as the carrier vehicles. Thereafter, a series of MWCNT‐Pt(IV) prodrugs have been developed. The first paradigm was Pt complex **10** which was entrapped within the inner cavity of MWCNT.^[26]^ Under the existence of deoxyguanosine monophosphate (dGMP), the MWCNT can release the active cisplatin under the reducing environment, thereby generating Pt‐dGMP adducts. The in vivo experimental results elucidated that the MWCNT‐Pt(IV) nanocomposite did not influence the cisplatin distribution but facilitated the accumulation of Pt(IV) species in certain organs, especially the lungs.^[27]^ Besides, as proved by the normal cytokine levels and histological investigations, the MWCNT did not cause any abnormal intrinsic immune response or inflammation in vivo. With the aim to enhance anti‐tumor performance and achieve precise delivery, a anthracycline‐based topoisomerase II (TOP2) inhibitor doxorubicin‐bearing Pt(IV) prodrug **11** was conjugated with integrin‐targeting cyclic peptide c(RGDfk)‐functionalized MWCNT delivery vehicle.^[28]^ Triggered by the cellular reductants, the MWCNT‐mediated Pt(IV) prodrug can simultaneously release doxorubicin as well as cisplatin to display superior cytotoxicity towards ovarian and endometrial tumor cells.

**FIGURE 6:**
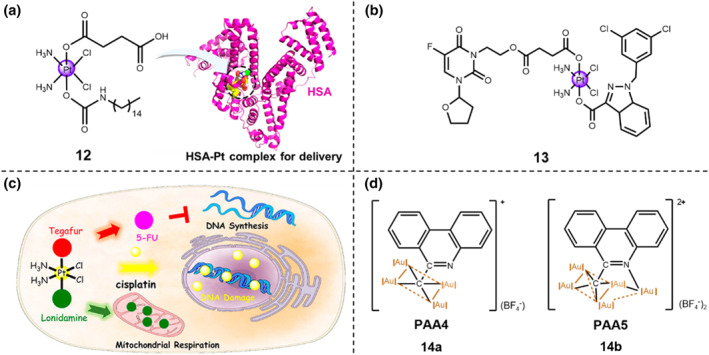
(a) Chemical structure of Pt(IV) prodrug **12** and lowest energy docked conformation of HSA‐Pt nanocomposite. Reproduced with permission: Copyright 2014, American Chemical Society.^[29]^ (b) Chemical structure of Pt(IV) prodrug PFL **13**. (c) Schematic illustration of redox‐activable Pt(IV) prodrug **13** and the subsequent cellular toxicity mechanism. Reproduced with permission: Copyright 2020, American Chemical Society.^[30]^ (d) Chemical structures of hypercarbon‐centered polynuclear Au(I) clusters PAA4 (**14a**) and PAA5 (**14b**).

To imitate the overall characteristics of a fatty acid, a series of *c*,*c*,*t*‐[Pt(NH_3_)_2_Cl_2_(O_2_CCH_2_CH_2_COOH)(OCONHR)] complexes were synthesized.^[29]^ Among those Pt(IV) prodrugs, potent complex **12** showed high binding affinity to HSA *via* non‐covalent bindings and displayed the strongest cytotoxicity and highest cellular uptake towards three malignant cancerous cell lines, A549, cisplatin‐sensitive A2780, and cisplatin‐resistant A2780cis (Figure 6a). The resulting Pt‐HSA adduct displayed improved cytotoxicity than that of cisplatin. Though the Pt‐HSA adduct experienced slow reduction by ascorbic acid, HSA endowed the adduct with excellent stability in human blood. Thus, HSA functions as the delivery vehicles for Pt(IV) complex **12**.

Recently, Mao's group developed a multifunctional Pt(IV) prodrug **13**, c,c,t‐[PtCl_2_(NH_3_)_2_(lnd)(tgf)], termed as PFL, for inhibition of the mitochondrial function and to de‐energize cancer cells (Figure 6b,c).^[30]^ PFL consists of cisplatin, tegafur (tgf), and lonidamine (Ind) and displays high selective anti‐proliferative activity towards triple negative breast cancer (MDA‐MB‐231) cells. The lonidamine, tethered with Pt(II) species through bidentate N,Ń chelating ligands, acts as the mitochondrial hexokinase inhibitor and triggers the mitochondrial apoptotic pathway. From the mechanistic perspective, complex **13** mainly localizes in the mitochondria and causes the reduction of mitochondrial membrane potential and inhibition of aerobic glycolysis and mitochondrial respiration. Furthermore, complex **13** leads to mitochondrial DNA damage and disturbs metabolism and transcription regulator activity, resulting in the apoptotic cell death by the mitochondrial pathway.

In the past decade, biologically active gold‐based metallodrugs have received increasing attention in the biomedical field. For example, Xiao et al. synthesized two GSH‐triggered hypercarbon‐centered polynuclear Au(I) clusters PAA4 (**14a**) and PAA5 (**14b**) through an intramolecular cyclization reaction of polymetalated precursors.^[31]^ The amino group in the precursor 2′‐((trimethylsilyl)ethynyl)‐[1,1′‐biphenyl]‐2‐amine (TEBA) was triggered by [(AuPPh_3_)_3_(μ_3_‐O)](BF4) ([Au_3_O]) to form a nucleophilic unit [NAu_3_]. Under the GSH‐overexpressed weakly acidic condition, the experimental results verified that the distinctive hypercarbon‐tetragold(I) multi‐center bonding in PAA4 and PAA5 ensured the stability, promoted the GSH‐activable prompt and synergistically released free Au^+^ ions. The Au(I) metallo‐prodrug can efficiently inhibit the thioredoxin reductase (TrxR) activity, induce a kinetical ROS generation, and trigger a pro‐oxidant response, thereby accelerating cell death by ferroptosis through the massive release of coordination unsaturated Au^+^ ions in human bladder cancer EJ cells. Consequently, these two Au(I) cluster metallo‐prodrugs displayed efficient cytotoxicity against bladder cancer cells and showed great inhibition effect in bladder tumor treatment. The above‐mentioned synergistic domino dissociation of Au(I) cluster metallo‐prodrugs provides new insights for the synthesis and application of metal cluster complexes.

## 3 EXOGENOUS‐STIMULI‐ACTIVATED METALLO‐PRODRUGS

Compared with the above‐mentioned endogenous triggers within the disease microenvironment, exogenous triggers, such as ultraviolet,^[32]^ visible light,^[33]^ near‐infrared (NIR) light,^[34]^ ultrasound,^[35]^ and radiation,^[36]^ can be exploited to activate the smart metal‐based prodrugs precisely and spatiotemporally. Thus, controlled release of metallo‐prodrugs can be attained by exogenous triggers, holding great potential for clinical applications with largely decreased toxicity and effects in future. Herein, anti‐cancer metallo‐prodrugs triggered by exogenous stimuli will be discussed and summarized in this section.

### 3.1 Light

As a stimulation in the design of exogenous‐stimuli‐activated metallo‐prodrugs, light has garnered substantial attention owing to its non‐invasive and precise spatiotemporal features.^[37]^ In general, there are two primary approaches for the construction of light‐activable metallo‐prodrugs, (1) direct photo‐activation of metallo‐prodrugs by photo‐induced structural transformation to generate the bioactive species, that is, metal‐based complexes or the released ligands; (2) The elevated level of cytotoxic ROS *via* photo‐irradiation of photosensitizers to cause cancer cell death. The second approach is photodynamic therapy for the cancer treatment.^[38]^ Nevertheless, photodynamic therapy can work only under the existence of O_2_ for current clinical photo‐sensitizers, probably limiting the biomedical applications in the hypoxic tumor microenvironment. The photo‐activable feature can be regulated by tuning the wavelength, exposure period, and power density of light. Despite the fact that the light in ultraviolet, visible, and NIR windows can be employed as stimuli for metallo‐prodrugs, only the NIR laser displays enhanced tissue penetration depth, superior accuracy, and excellent spatio‐temporal resolution.

#### 3.1.1 Ultraviolet‐light‐activable metallo‐prodrugs

**FIGURE 7:**
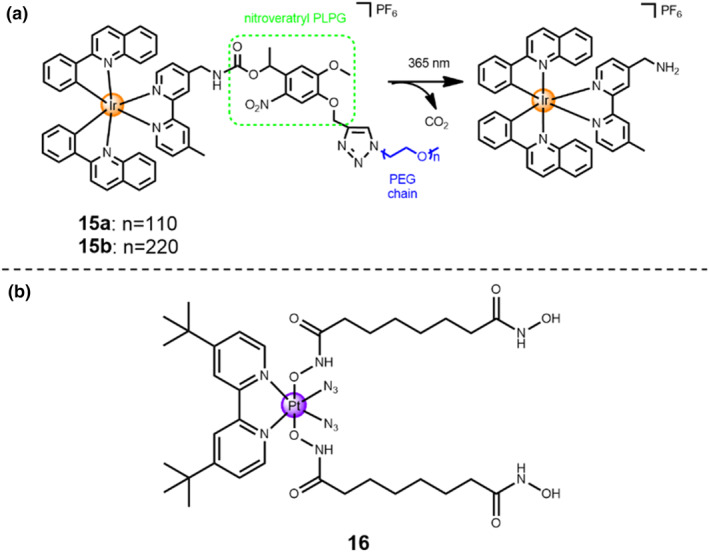
(a) Schematic mechanism of ultraviolet‐light‐responsive Ir(III) prodrugs **15 a**‐**b**. (b) Chemical structure of Pt(IV) complex **16**.

To minimize the cytotoxicity of Ir(III)‐based metallo‐prodrugs under dark conditions, Lo's group developed light‐responsive pegylated Ir(III) complexes **15a**‐**b** (Figure 7a).^[32a]^ In this study, a nitroveratryl linker functioned as a photo‐cleavable protecting moiety between the Ir(III) complex and the polyethylene glycol (PEG) chain. The PEGylated modification was proved to greatly reduce the cytotoxicity of luminescent transition metal compounds. Under the dark condition, the PEGylated Ir(III) compounds **15a** and **15b** showed lower cytotoxicity against HeLa cells compared to that of Ir(III) complex alone. After 20‐min 365‐nm light irradiation, the two compounds **15a** and **15b** showed a high photocytotoxicity against HeLa cells, with superior phototoxicity indexes of 25.9 for **15a** and 13.5 for **15b**, respectively.

In another study, Kasparkova and co‐workers developed a photo‐stimuli‐triggered Pt(IV)‐diazido complex **16**.^[32b]^ In this study, two suberoyl‐bis‐hydroxamic acid (SubH) as axial ligands can function as the histone deacetylase (HDAC) inhibitors, resulting in the enhanced inhibition of cancer cell growth and proliferation. Hence, Pt(IV) complex **16** can act as a photo‐active prodrug with dual functionalities: the high cytotoxicity of active Pt(II) drug as well as the improved inhibitory effect of the released axial SubH units. When exposed upon ultraviolet (365 nm, 4.3 mW/cm^2^) or visible light (458 nm, 65 mW/cm^2^) irradiation, the Pt(IV) complex **16** displayed strong binding with DNA, but it was kinetically inert under dark condition. Additionally, under the existence of cellular reductants, for example, ascorbic acid and GSH, it possessed superior stability in the dark. It was worth mentioning that the photo‐active cytotoxicity of Pt(IV) complex **16** in both cisplatin‐sensitive A2780 and cisplatin‐resistant A2780cis cancer cell lines was remarkably higher than that of cisplatin alone and the derivative of Pt(IV) complex **16** consisting of biologically inactive axial ligand. Consequently, a combined mechanism was proved for the complex **16**, that is, the inhibition of HDAC by SubH moieties as HDAC inhibitors, the steric blockage of RNA polymerization, and interferences of DNA ascribed to the active Pt(II) drug strongly binding with DNA and the formation of DNA adducts.

#### 3.1.2 Visible‐light‐activable metallo‐prodrugs

**FIGURE 8:**
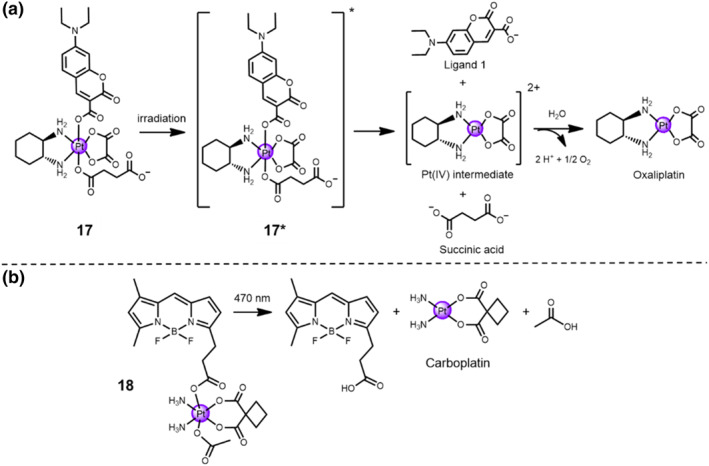
(a) The proposed responsive mechanism of coumaplatin **17** by 450 nm blue light in PBS buffer. (b) Photo‐responsive BODIPY–Pt prodrug **18**.

The visible light spectrum is the electromagnetic spectrum segment that can be detected by human eye, ranging from 400 to 700 nm, including green, blue, and red light, *etc*. Zhu and the co‐workers has developed a blue‐light‐activable coumarin‐modified oxliplatin‐based Pt(IV) complex **17**, namely as coumaplatin.^[33a]^ When exposed upon blue light irradiation (450 nm), the Pt(IV) complex **17** could be reduced to oxaliplatin through intermolecular electron transfer process with water solvent acting as the electron donor during photo‐activation period, forming O_2_ as byproducts (Figure 8a). Unexpectedly, this distinct nucleolus‐targeting Pt(IV) prodrug **17** exhibits remarkably ability, that is, overcoming the drug‐resistant issues, inducing cell senescence and immunogenic cell death (ICD) along with T cell proliferation, and provoking anti‐tumor activity. After blue light irradiation treatment, the primary hallmarks of ICD were detected to prove the activation of immune system, including the elevated calreticulin levels on the cellular membrane, the high‐mobility group box 1 protein release, and adenosine triphosphate (ATP) secretion. 4,4‐Difluoro‐4‐bora‐3a,4a‐diaza‐*s*‐indacene dyes, namely as BODIPY dyes, tend to be strongly ultraviolet/visible‐light‐absorbing small molecules that emit relatively sharp fluorescence peaks with high quantum yields. In other studies, the same group also developed a green‐light‐activable Pt(IV) prodrug **18** conjugated to a BODIPY moiety as a photo‐absorber (Scheme 30).^[33b,33c]^ The 470‐nm green light irradiation treatment facilitated the electron transfer process from the reducing agent to the metal center, making it reduced to carboplatin. Notably, the cytotoxicity of the Pt(IV) prodrug **18** was higher against MCF‐7 cells and displayed enhanced cell killing effect than that of carboplatin after green light exposure, with IC_50_ value = 15.7 ± 1 μM for Pt(IV) prodrug **18** and IC_50_ value = 642.6 ± 51.4 μM for carboplatin, respectively. When exposed to green light, the cytotoxicity of Pt(IV) prodrug **18** was 39 times higher than that of carboplatin alone. The phototoxicity index (PI) of Pt(IV) prodrug **18** is calculated as 11 against MCF‐7 cells, while it displays negligible toxicity under dark conditions, with IC_50_ value = 173.4 ± 8.8 μM.

**FIGURE 9:**
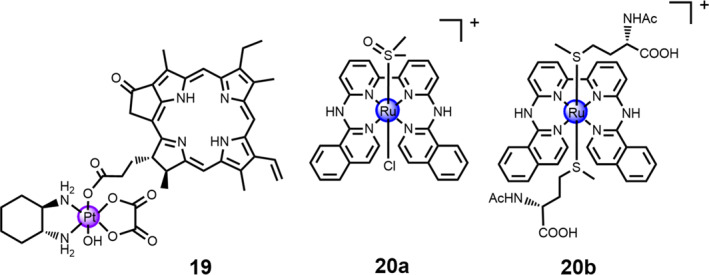
Chemical structures of Pt(IV) prodrug **19** (phorbiplatin) and Ru(II)‐based prodrugs **20a**‐**b**.

Recently, Zhu and co‐workers has developed a red‐light‐responsive oxaliplatin‐based Pt(IV) prodrug **19**, namely as phorbiplatin [Pt(DACH)(PPA)(OH)(ox)], through flanking pyropheophorbide a (PPA) at the axial position of Pt(IV) prodrug **19** (Figure 9).^[33d]^ Notably, phorbiplatin showed a mild cytotoxicity in dark, but it displayed a remarkable cell inhibition activity against MCF‐7 cells, which was >1780 times more cytotoxic than that of oxaliplatin after 48‐h incubation of phorbiplatin. The mechanistic investigations elucidated that the PPA axial ligand promotes the electron transfer from cellular reductants to the Pt(IV) center when treated with red light irradiation (660 nm, 100 mW/cm^2^), thereby facilitating the release of active oxaliplatin leading to DNA damage, accelerated DNA generation and apoptotic cell death. Additionally, the in vivo experimental results revealed that phorbiplatin showed excellent anti‐tumor effects with a 67% inhibition rate on the tumor volume of 4T1‐xenograft mice model compared to that of the control group. Taken together, the controllable activation and remarkable anti‐tumor activity of Pt(IV) prodrug **19** open a new door for the construction of red‐light‐activable platinum‐based prodrugs to minimize the side effects and solve the drug‐resistant problems of the conventional platinum drugs.

In recent decades, Ru(II) complexes, as promising photo‐caging complexes for light‐responsive chemotherapy, have attracted much attention.^[37]^ The photo‐substitution of Ru(II) complexes can release biologically active units when exposed upon visible light irradiation. Based on a tetrapyridyl biqbpy ligand and two trans monodentate sulfur ligands, two photo‐activable Ru(II)‐based prodrugs **20a** and **20b** were successfully synthesized (Figure 9).^[33e]^ After treated with 520‐nm green light irradiation at a dose of 75 J/cm^2^, the trans monodentate sulfur ligands within both Ru(II) complexes underwent photo‐substitution with water. The Ru(II) complexes showed remarkably low quantum yields of ^1^O_2_ (∼1–2%) and a relatively low cytotoxicity under dark conditions. Compared to the dark control group, when exposed upon 520‐nm green light irradiation (75 J/cm^2^), an up to 22‐fold enhancement of in vitro cytotoxicity in A549 and A431cell lines was detected with effective concentration <1 μM. The confocal laser microscopy scanning and flow cytometry analysis results verified that Ru(II) complexes induced apoptosis in A549 cells.

#### 3.1.3 Near‐infrared‐light‐activable metallo‐prodrugs

**FIGURE 10:**
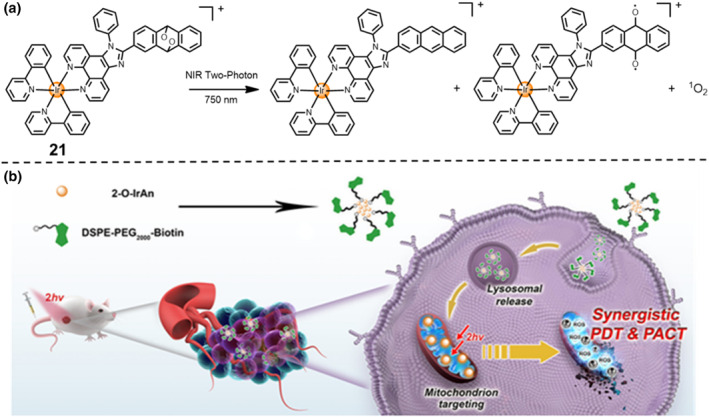
(a, b) Mechanistic scheme of the action of the Ir(III) prodrug **21** by synergistic photodynamic therapy/photo‐activated chemotherapy. Reproduced with permission: Copyright 2022, American Chemical Society.^[34a]^

The efficacy of photodynamic therapy is hindered by many factors, for example, hypoxic tumor microenvironment, poor targeting ability and deficient delivery of anti‐cancer drugs. To address the above‐mentioned problems and enhance the curative efficacy, Chao's group designed and synthesized a novel photosensitized cyclo‐metalated Ir(III) prodrug **21** namely as 2‐O‐IrAn.^[34a]^ Exposed upon the two‐photon NIR‐I irradiation, the triggered 2‐O‐IrAn can simultaneously release a cytotoxic Ir(III) complex (2‐IrAn), an alkoxy radical, and ^1^O_2_ (Figure 10a). Under hypoxic conditions, the 2‐O‐IrAn displayed excellent phototoxic effects both in the A549 cancer cells and MCTSs at the nanomolar scale, with a IC_50_ = 60 nM and a PI = 690.3. The mechanism investigation elucidated that the Ir(III) complex was mainly localized in the mitochondria, leading to the loss of mitochondrial membrane potential and thereby inducing cell death by the apoptotic pathway (Figure 10b). To enhance the selective cellular uptake, the Ir(III) complex was encapsulated with biotin‐modified polymer and generated the DSPE‐PEG‐Biotin@2‐O‐IrAn nanoparticles. Notably, exposed upon two‐photon irradiation at 750 nm, the as‐prapared nanoparticles can completely eradicate the subcutaneous tumor in the A549 tumor‐bearing nude mouse with a single treatment. Taken together, this study employs a synergistic bimodality therapy with photodynamic therapy and photoactivated chemotherapy to address the hypoxia issues in cancer treatment.

**FIGURE 11:**
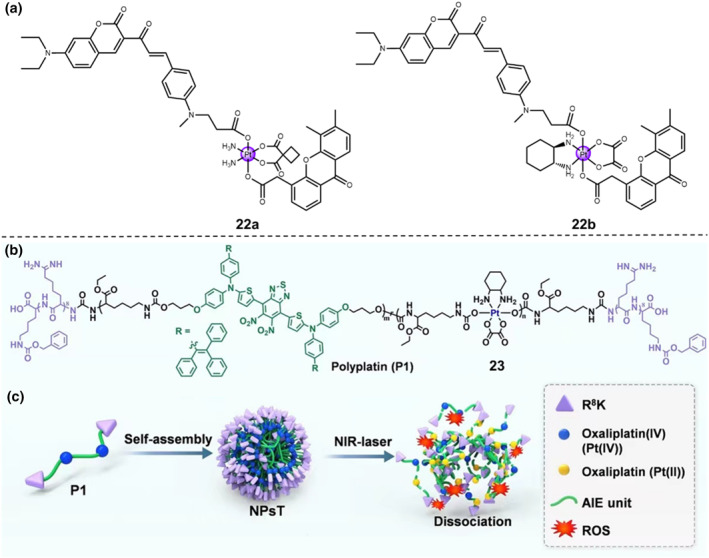
(a) Chemical structures of Pt **22a** and **22b**. (b) Chemical structure of the functionalized polyplatin **23** (P1), which consists of the Pt(IV) complex, the aggregation‐induced emission (AIE) moiety, and terminal targeting peptides R^8^K. (c) In an aqueous solution, the polyplatin can self‐assemble into NPsT. The dissociation of the NPsT is triggered by the NIR‐I laser. Reproduced with permission: Copyright 2022, WILEY‐VCH.^[34c]^

Recently, Zhu's group developed a pair of NIR‐light‐activated photooxidants, carboplatin‐ and oxaliplatin‐based Pt(IV) complex (Pt **22a** and Pt **22b**, Figure 11a), by introducing 5,6‐dimethylxanthenone‐4‐acetic acid (DMXAA) unit and the coumarin‐based photosensitive ligand moiety at the axial positions.^[34b]^ These as‐prepared Pt(IV) complexes were endowed with strong metal‐enhanced photooxidation ability in a controllable oxygen‐independent manner, showing improved cellular uptake selectivity and boosting photodynamic therapy activity. The Pt(IV) prodrugs were localized in the endoplasmic reticulum and remained inactive under dark conditions, while upon 880‐nm NIR‐I laser irradiation, they simultaneously released the corresponding active Pt(IV) drugs and the axial ligands, resulting in the severe oxidative stress and imbalanced pH homeostasis to damage cancer cells and thereby inducing cell death by non‐classical necrosis pathway. Notably, the in vivo experimental results revealed that Pt **22a** photo‐oxidant suppressed the tumor growth, inhibited the tumor metastasis, and subsequently activated the immune system in tumor microenvironment, which were attributed to the superior anti‐tumor and anti‐metastatic effects of Pt **22a** photo‐oxidant (carboplatin‐based Pt).

In another study, Xiao and Karges groups have reported the incorporation of aggregation‐induced emission (AIE) unit with absorption in the red region and an oxaliplatin unit into NIR‐responsive polyplatin **23** (Figure 11b).^[34c]^ Building upon the amphiphilic characteristic of the polymeric backbone, the polymer chains can undergo a self‐assembly process into spherical nanoparticles (NPsT) (Figure 11c). Because of the improved permeability, retention ability, and the terminal functionalization with nucleus‐targeting peptides R^8^K, the NPsT can selectively accumulate in the nucleus of tumor cells. The NPsT can maintain stability and be therapeutically inactive under the dark conditions. When exposed by the 808‐nm NIR‐I laser, the Pt(IV) complex will reduce into oxaliplatin and the NPsT will undergo dissociation, leading to the release of the polymeric ligands at the axial positions. The intravenous injection of the NPsT into 4T1 tumor‐bearing BALB/c mice showed a remarkable tumor inhibition effect and the increased survival rate of the mouse models.

### 3.2 Ultrasound

Although significant advances have been realized by light‐activated metallo‐prodrugs in cancer chemotherapy, it is still a great challenge to ablate tumors treated by exogenous‐stimuli‐activated metallo‐prodrugs with deep tissue penetration. Nowadays, sonodynamic therapy is a novel modality for cancer treatment based on the photodynamic therapy.^[39]^ In comparison with photodynamic therapy, which shows shallow tissue penetration, ultrasound possesses abundant edges, for example, deep tissue penetration, spatiotemporal accuracy, and less‐invasive treatment features. Additionally, sonodynamic therapy is more facile and readily available in medical settings. When subjected to the ultrasound radiation, the Pt(IV) complex can maintain stability in a physiological environment and undergo a reduction process to the active Pt(II) species under the existence of a sonosensitizer.

**FIGURE 12:**
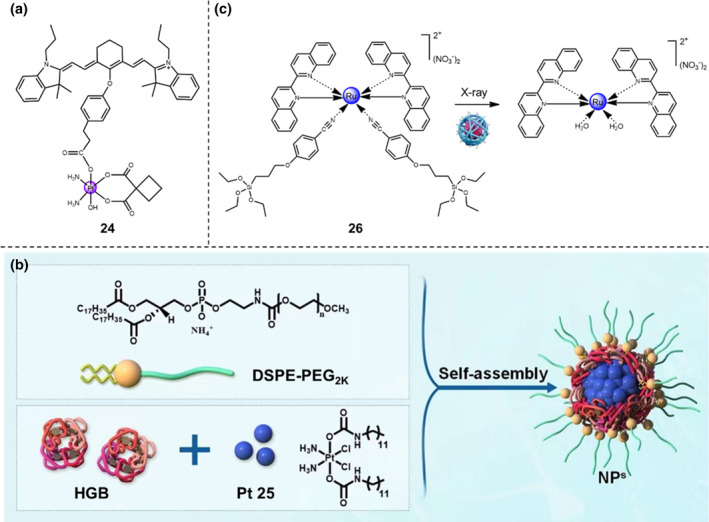
(a) Chemical structure of cyaninplatin, Pt(IV) complex **24**. (b) Illustration of self‐assembly of HGB with a hydrophobic Pt(IV) complex (**Pt 25**) into nanoparticles. Reproduced with permission: Copyright 2023, WILEY‐VCH.^[35b]^ (c) Schematic illustration of degradation of Ru‐Si **26** exposed by X‐ray radiation. Reproduced with permission: Copyright 2023, WILEY‐VCH.^[36]^

Recently, Zhu and the co‐workers have developed an ultrasound‐responsive theranostic Pt(IV) complex **24** by anchoring heptamethine cyanine sonosensitizer (IR780) at the axial position, namely as cyaninplatin (Figure 12a).^[35a]^ When subjected to the sono‐activation, the IR780 moiety of the cyaninplatin facilitates the electron transfer process, thus the mitochondria‐targeted cyaninplatin displays significant mitochondrial DNA damage and enhanced cell killing effect against 4T1 cells. Owing to the combined effects of released carboplatin, the rapid generation of ROS, and the exhaustion of intracellular reductants, that is, nicotinamide adenine dinucleotide (NADH) and GSH, the cyaninplatin addresses the drug‐resistance issue. This process ablates the malignant 4T1 cells by the combined mechanism of both paraptosis and eliciting of ICD, greatly enhancing the anti‐tumor effects. Building upon the high‐resolution ultrasound, photoacoustic and NIR‐I optical trimodal imaging, cyaninplatin prodrug is a promising theranostic nanoplatform with suitable biocompatibility and excellent curative efficacy. This study exploits the practical function of ultrasound for activation of the anti‐tumor Pt(IV)‐based prodrugs in a spatiotemporally and precisely controllable manner, achieving the eradication of deep tumor tissues and widening the biomedical application of Pt‐based metallo‐prodrugs.

In another study, Xiao and Karges groups have reported the selective reduction of ultrasound‐responsive Pt(IV) metallo‐prodrugs for eradicating deep‐seated tumors.^[35b]^ Clinical results have demonstrated that the ultrasound radiation shows much deeper tissue penetration (around 10 cm) compared to that of NIR‐I light, showing great potential in the treatment of large or deep‐seated tumors. A smart ultrasound‐responsive anti‐cancer agent, Pt(IV) prodrug **25**, was successfully synthesized to improve the therapeutic efficacy in a multi‐modal approach. To improve water solubility and enhance sono‐sensitivity, the Pt **25** was encapsulated within nanoparticles by the biocompatible sonosensitizer hemoglobin (HGB) (Figure 12b). The experimental results displayed that the nanoparticles underwent rapid dissociation when exposed to the ultrasound radiation, thereby triggering cell death in the apoptotic pathway. To evaluate the biological efficacy, the nanoparticles were further assessed in CT26 tumor‐bearing mice. Intravenous injection of the nanoparticles achieved specific accumulation at the CT26 tumor area. Remarkably, when subjected to the ultrasound radiation, the tumors were completely eradicated in almost all the mouse models, verifying the effectiveness of this strategy for cancer treatment.

### 3.3 Radiation

Recently, Ruan et al. proposed a general approach for the radiation‐activated metallo‐prodrugs through nanosurface energy transfer (NSET).^[36]^ In this study, the core‐shell nanosystem consisting of gold nanoclusters (GNC) and Ru‐based organic‐inorganic hybrid coatings is termed as Ru‐GNC. Exposed by X‐ray radiation, the Ru‐Si **26** underwent degradation and the active Ru(II) agents were released by a controlled NSET process, which involves the transfer of photoelectron energy from the radiation‐excited Ru‐GNCs to the Ru‐based hybrid coating (Figure 12c). Compared with the conventional radiation‐activated prodrugs, these ultrasmall Ru‐GNCs guarantee the abundant reactive species which tend not to be quenched in the tumor microenvironment. Moreover, this NSET‐based mitochondria‐targeting nanosystem can destroy the respiratory chain exposed by X‐ray radiation, causing radio‐sensitization by the formation of sufficient ROS. As a result, through the integration with programmed cell death‐ligand 1 (PD‐L1) checkpoint blockade, Ru‐GNC‐mediated radiochemotherapy elicits ICD, leading to excellent curative efficacy. The experimental results prove that this smart NSET approach achieves a breakthrough in the development of radiation‐activated nanosystems for metallo‐prodrug‐directed radiochemotherapy.

## 4 DUAL‐, AND MULTI‐STIMULI‐ACTIVATED METALLO‐PRODRUGS

Owing to the intricate tumor microenvironment, single‐stimuli‐responsive systems probably cannot meet the various demands simultaneously, for example, precisely spatiotemporal drug release and delivery needs. As an alternative, dual‐ and multi‐stimuli‐responsiveness systems with two or three internal or external stimuli have been developed for the complex pathological conditions.^[40]^ Herein, metallo‐prodrugs with dual‐ and multi‐responsiveness to different types of triggers are summarized in this section.

### 4.1 Redox/light‐activable metallo‐prodrugs

**FIGURE 13:**
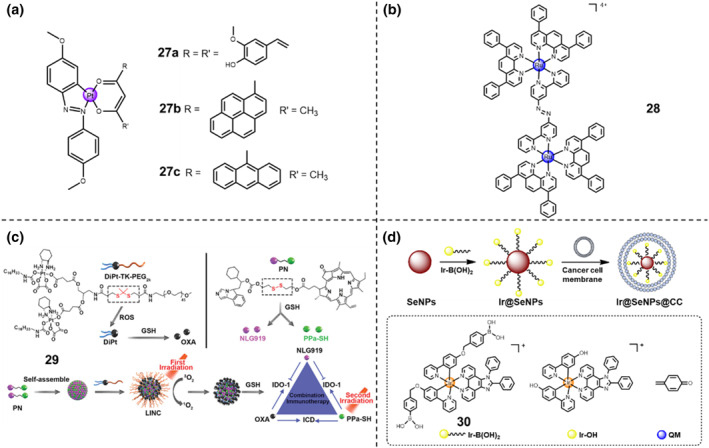
(a, b) Chemical structures of redox/light‐activable (a) Pt(II) complexes **27a**‐**c** and (b) dinuclear ruthenium(II)‐azo complex **28**. (c) Scheme of NIR‐I laser induced light‐inducible nano‐cargo (LINC) for self‐amplified strategy and chemoimmunotherapy. Reproduced with permission: Copyright 2019, WILEY‐VCH.^[43]^ (d) Synthetic scheme and mechanism of action of Ir@SeNPs@CC nanocomposite for synergistic chemotherapy and two‐photon photodynamic therapy.

Pt(II) complexes **27a**‐**c** of monoanionic β‐diketonates were developed as GSH/light dual‐activable prodrugs (Figure 13a).^[41]^ As the leaving groups in the Pt(II) complexes, β‐diketonates possess the ability for ROS generation under visible light irradiation (400–700 nm), holding the potential as the photosensitizer agents for photodynamic therapy. The cyclometalated Pt(II) unit remains stable under cellular medium. Consequently, GSH can solely substitute the monoanionic O,O‐donor β‐diketonate ligands, ensuring the slow release of β‐diketonate. Hence, under visible light conditions (400–700 nm, Luzchem photoreactor, 10 J/cm^2^), the Pt(II) complexes displayed photocytotoxicity in HaCaT keratinocytes with IC_50_ value of 8–14 μM, owing to the accelerated ROS formation from the leaving β‐diketonate ligands. Under the dark conditions, the Pt(II) complexes displayed a negative cytotoxicity with IC_50_ value of ∼60 μM. Taken together, this GSH/light dual‐activable feature was of great importance for triggering the Pt(II) complex **27a**‐**c**.

Later, Chao's group designed and synthesized a dinuclear ruthenium(II)‐azo complex **28** (Figure 13b), functioning as a photosensitizer which was responsive under the existence of intracellular GSH.^[42]^ Upon GSH addition in PBS solution (0–50 μM), the changes in the absorption spectra of complex **28** (10 μM) were in line with the GSH absorption titration study, where 50‐fold enhanced peak intensity of the photoluminescence emission at 640 nm can be detected along with the increased concentration of GSH. The Ru‐azo complex displayed large mitochondria accumulation within Hela cells, indicating that organelle mitochondria were the primary targets of the Ru‐azo complex. Moreover, the Ru‐azo complex displayed a small dark cytotoxicity with IC_50_ value > 70 μM towards Hela, A549, and LO2 cells, while under the 450 nm light irradiation (20 mW/cm^2^, 15 min), the Ru‐azo complex displayed a 14‐fold higher phototoxicity with IC_50_ value = 5 μM towards the above three cancer cell lines. In 3D MCTS of Hela cells, the Ru‐azo complex was inactive under dark (IC_50_ > 100 μM) while the Ru‐azo displayed a 19‐fold enhancement phototoxicity (IC50 = 5.71 μM) against MCTS under two‐photon NIR laser irradiation (810 nm, 100 mW, 80 MHz, 100 fs).

By covalent or non‐covalent conjugation, Pt(IV) prodrugs have achieved effective loading into different drug delivery materials. The advances in drug delivery materials endow them with more functions. Nowadays, the design and construction of a delivery system with synergistic therapy abilities can be achieved. For instance, with the aim to prevent the cellular reductants during blood circulation, a Pt(IV) prodrug **29** with a cloak barrier was developed by a nanovehicle approach to solve the instability and deactivation problems.^[43]^ Feng et al. developed a GSH/light dual‐inducible oxaliplatin‐based prodrug, which was PEGlyted with an ROS‐sensitive thioketal spacer (Figure 13c). Then, the PEGlyted amphiphilic prodrug was loaded with a disulfide bond‐conjugated heterodimer molecule of IDO‐1 inhibitor NLG919 (PN)‐photosensitive pheophorbide A (Ppa). Exposed by the NIR‐I laser, the Ppa unit facilitated the ROS formation to cleave the thioketal spacer, thereby resulting in the PEG shell detachment and the prolongation of tumor retention period of this oxaliplatin‐based nanosystem. After the first wave of NIR‐I laser irradiation, this oxaliplatin‐based nanosystem was activated by the elevated intracellular GSH in tumor microenvironment. The oxaliplatin restored by the GSH greatly suppressed the tumor progression and induced immunogenicity, while the released NLG9219 overcame the immunosuppressive tumor microenvironment by deactivating the IDO‐1 activity. Overall, this oxaliplatin‐based chemotherapy combined with the NLG919‐mediated immunotherapy displayed the best anti‐tumor therapeutic efficiency compared with other single mode of therapy groups.

Melanoma is one of the most invasive and deadliest types of skin cancer. In addition to the conventional clinical techniques, the utilization of photodynamic therapy against melanoma has gained considerable attention. Most of iridium compounds are activated with ultraviolet or visible light, limiting the utilization of subcutaneous tumor treatment. To address these issues, Chao and the co‐workers reported a H_2_O_2_‐responsive cancer cell membrane‐coated Ir(III) complex **30** (Ir‐B(OH)_2_)‐modified selenium nanoparticles, namely as Ir@SeNPs@CC, for synergistic chemotherapy and photodynamic therapy (Figure 13d).^[44]^ Once triggered by the H_2_O_2_ activation, the nanocomposite prodrug quickly underwent dissociation, resulting in the release of cyclometalated Ir(III) complex (Ir‐OH), acting as the photosensitizer, and the methylquinone (QM) and Se nanoparticles (SeNPs), acting as the GSH scavengers and chemotherapeutic agents. The depletion of intracellular GSH improved the chemical sensitivity of A375 cells for efficient photodynamic therapy. The Ir@SeNPs@CC displayed enhanced chemical selectivity, cellular membrane permeability, and pharmacokinetic effect for A375 cells. Exposed upon the two‐photon irradiation at 730 nm, the Ir@SeNPs@CC nanocomposites could identify and almost completely ablate the A375 xenograft within a A375 tumor‐bearing nude mice by the dual‐modal therapy combined with chemotherapy and NIR photodynamic therapy.

### 4.2 pH/light‐activable metallo‐prodrugs

Some pH/light dual‐stimuli activable metal‐based complexes are developed as prodrugs to diminish the off‐target by effects and selectively kill malignant tumor cells. For instance, the Ru(II) complex **31** with a pH‐sensitive ligand 6,6′‐dihydroxy‐2,2′‐bipyridine was designed and synthesized.^[45]^ It would undergo photodissociation from the metal center under a slightly acidic environment in cancer cells. Under dark conditions, the Ru(II) complex does not exhibit obvious cytotoxicity towards Hela cells, while the cell viability largely decreases to 47% with a IC_50_ = 88 μM against Hela cells exposed upon 450 nm light irradiation for 1 h. Later, Qu et al. constructed a pH/light dual‐trigger‐responsive Ru(II) complex **32**.^[46]^ The slightly acidic microenvironment in cancer cells activates the Ru(II) prodrug through weakening the bond between Ru metal center and the phenanthroline ligand. Subsequently, there undergoes the photo‐dissociation under 450 nm light irradiation, followed by the release of the active Ru complex. The experimental results verify the off‐target side effects of the Ru complex are minimized, with IC_50_ = 4 μM against MCF‐7 cancer cells under 450 nm light irradiation, which are even comparable to the cytotoxicity of cisplatin with IC_50_ < 2 μM against MCF‐7 cancer cells with the same treatment.

**FIGURE 14:**
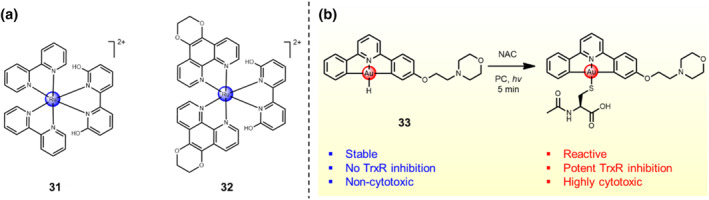
(a) Chemical structures of pH/light‐activable Ru(II) complex **31**–**32**. (b) Photocatalytic transformation of Au prodrug **33** into the resulting Au(III)–S adduct under the existence of a 100‐fold excess of NAC.

Photocatalysis has been applied in localized prodrug activation in the biomedical field. Nevertheless, light with high power density is needed without providing controlled selectivity and high efficiency. Recently, Zou and co‐workers developed a gold‐based prodrug **33** (Au 1d) modified with a pH‐sensitive morpholine unit and proposed that ion pairing between the prodrugs and photo‐catalysts can greatly enhance the photo‐activation efficiency and enable the red‐light‐activated tumor‐targeting ability (Figure 14b).^[47]^ The modification with morpholine moiety enable prodrug **33** to undergo hydrolysis in aqueous solvent, generating a hydrolyzed cationic form that strongly interacts with Eosin Y (EY) and Rose Bengal (RB), the anionic photosensitizers. In the UV‐Vis‐NIR absorption spectra, a significant bathochromic shift was detected in the spectrum of “EY + 1d” mixture with a long tail within the NIR‐I region (750–800 nm). Consequently, an excellent photo‐activation efficiency of Au prodrug **33** by EY or RB exposed upon low‐energy red light irradiation was detected, resulting in a controlled release of active gold species in the living *E*. *coli* cells and inhibition of TrxR activity. The activation mode was observed by the emitted red fluorescence of a gold‐specific biosensor, namely the GolS‐mCherry system. Notably, the morpholine unit (pKa = 6.9) endows Au prodrug **33** with pH‐responsive trait and Au prodrug **33** can generate ionic interaction with EY under a weakly acidic environment, thereby realizing red‐light‐activable gold‐based prodrug with tumor targeting ability in vitro and in vivo. The similar above‐mentioned phenomena were also detected in morpholine (amine)‐containing clinic drugs, photocages and the precursors of reactive labeling intermediates; the ion‐pairing strategy holds the potential for expanding the repertoire of chemical tools in the biomedical field.

### 4.3 Redox/pH‐activable metallo‐prodrugs

Before being reduced into the active Pt(II) species by cellular reductants like GSH and ascorbic acid, Pt(IV) prodrugs are usually inert and widely used for minimizing the severe by effects. Another strategy to enhance the curative efficiency of Pt(II) drugs is to load them into a delivery carrier, through this way the precise delivery as well as controlled release of the drugs activated by the endogenous stimulations can be achieved. Building upon this strategy, stimuli‐activable drug delivery systems involving Pt(IV) prodrugs has been investigated to prepare dual‐stimuli‐responsive metallo‐prodrugs.

**FIGURE 15:**
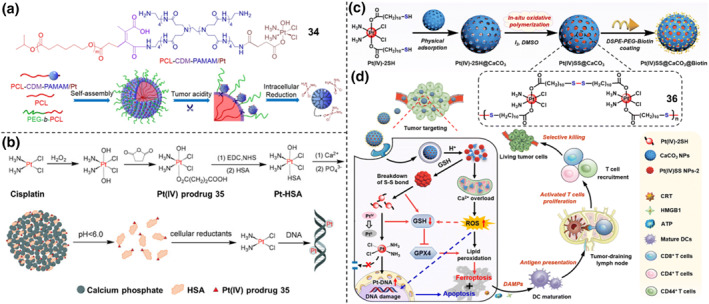
(a) Chemical structure of clustered PCL‐CDM‐PAMAM/Pt **34** and the pH/redox dual‐activable mechanism of iCluster/Pt in tumor microenvironment. Reproduced with permission: Copyright 2016, National Academy of Sciences.^[48]^ (b) Synthetic scheme of the Pt–HSA/CaP nanocomposites and the release of active cisplatin in response to low pH and cellular reductants. Reproduced with permission: Copyright 2015, WILEY‐VCH.^[49]^ (c, d) Schematic illustration of (c) physical adsorption and oxidative polymerization of a cisplatin‐based prodrug **36**, termed Pt(IV)SS@CaCO_3_@Biotin, and (d) Pt(IV)SS@CaCO_3_@Biotin‐induced chemoimmunotherapy by releasing cisplatin drug, producing ROS and exhausting GSH to stimulate synergistic anti‐cancer effect of apoptosis and ferroptosis. Reproduced with permission: Copyright 2021, Elsevier.^[50]^

For example, Li et al. developed a dual pH/redox‐activated clustered nanoparticles, Pt(IV) prodrug‐conjugated poly(amidoamine)‐graft‐polycaprolactone (namely as PCL‐CDM‐PAMAM) (Figure 15a).^[48]^ Specifically, the PCL‐CDM‐PAMAM was selected to link the Pt(IV) prodrug **34**. The PCL‐CDM precursor was conjugated with PAMAM‐Pt by acid‐labile amide bonds, which experienced cleavage to release PAMAM/Pt under a weakly acidic environment. This pH‐instable clustered nanoparticle containing Pt(IV) prodrug (iCluster/Pt), obtained by the molecular assembly of PCL‐CDM‐PAMAM with PCL homopolymer and poly(ethylene glycol)‐b‐poly(ε‐caprolactone) (PEG‐*b*‐PCL) copolymer possesses an average diameter of ∼100 nm, which is suitable for long blood circulation and tumor vascular extravasation through EPR effect. As a result, iCluster/Pt displayed accelerated intracellular drug release of PAMAM/Pt(IV) prodrug dendrimers (∼5 nm) under acidic pH, enhancing the cell internalization and better drug accumulation at tumor site of BxPC‐3 tumor‐bearing nude mice which was seven‐fold larger drug concentration than that of cisplatin alone and PAMAM/Pt. Subsequently, the internalized dendrimer Pt(IV) complexes was intracellularly reduced to release active cisplatin to kill A549R cancer cells, displaying 95% anti‐tumor effect. Compared with the pH‐stable counterpart (Cluster/Pt), the iCluster/Pt treatment exhibited enhanced survival time in a metastatic 4T1 orthotopic tumor model.

In addition to the common polymers, biomacromolecules can act as delivery carriers for Pt(IV) prodrugs to develop smart pH/redox dual‐activable drug delivery systems. One typical paradigm was the development of a biocompatible hybrid nanoparticle consisting of HSA and calcium phosphate (CaP) for Pt(IV) complex (Pt‐HSA/CaP) (Figure 15b).^[49]^ HSA, a widely‐used drug carrier, possess the ability to protect Pt(IV) complexes from premature reduction before cellular uptake and enhance the targeting ability to malignant tumor tissues.^[29]^ Under the weakly acidic environment, CaP can be degraded for the release of Pt‐HSA from the conjugate. The Pt(IV) prodrug **35** was conjugated to HSA to produce a Pt‐HSA adduct. This hybrid Pt‐HSA/CaP nanoparticle with size of ∼100 nm was constructed by connecting the Pt‐HSA complex with CaP. Notably, Pt‐HSA/CaP displayed great stability under neutral media, while it showed responsiveness to weakly acidic media (endosomes or lysosomes, pH = 4.0–6.0) once it was internalized into cells by endocytosis, and then the Pt‐HSA complex was completely released. Additionally, the Pt‐HSA complex can act as the Pt(IV) prodrug of cisplatin, show responsive to the cellular reductants and then release cisplatin, which subsequently strongly binds to DNA. This pH/redox dual‐activable Pt‐HSA/CaP nanoparticle displayed greater cytotoxicity than that of solely cisplatin and the Pt(IV) prodrug **35** to five malignant tumor cell lines, that is, MDA‐MB‐231, HeLa, HepG2, A549, and MCF‐7 cells.

Recently, Chao's group have reported a thiol‐functionalized Pt(IV)‐based complex **36**, which was physically absorbed into the pore‐confined spaces of supramolecular CaCO_3_ nanoparticles and subsequently covalently linked by in situ oxidative polymerization to form disulfide (‐S‐S‐) bonds within Pt(IV) complex aggregates, namely as Pt(IV)SS@CaCO_3_ (Figure 15c,d).^[50]^ To improve the water solubility and targeting ability towards tumor, the CaCO_3_ surface was modified with DSPE‐PEG_2000_‐Biotin (Pt(IV)SS@CaCO_3_‐Biotin). After Pt(IV)SS@CaCO_3_‐Biotin entered the cisplatin‐resistant non‐small lung cancer cells (A549R), it rapidly underwent degradation into Ca^2+^ ions and Pt(IV) aggregated in a slightly acidic microenvironment. Subsequently, the ‐S‐S‐ bonds within Pt(IV) aggregate underwent breakdown to form thiols, and simultaneously cisplatin was released under the activation of intracellular GSH. This process induced multimodal activities involving Ca^2+^ overload in mitochondria, GSH exhaustion, nuclear DNA platination by cisplatin, accelerated ROS formation, and the accumulation of lipid peroxides on cellular membranes, triggering cancer cell death by a hybrid pathway of apoptosis, ferroptosis, and ICD. At last, the in vivo experimental results proved that the Pt(IV)SS@CaCO_3_‐Biotin can induce ICD in the immunocompetent C57BL/6J mice vaccinated with the pre‐treated Lewis lung carcinoma (LLC) cells, providing new insights for the novel synthetic strategy for chemoimmunotherapy agents in the clinical translation and the treatment of drug‐resistant, distant and metastatic tumors.

### 4.4 Light/enzyme‐activable metallo‐prodrugs

**FIGURE 16:**
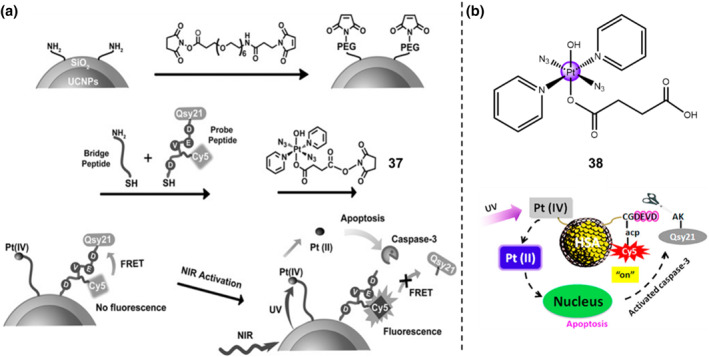
(a) Scheme of Caspase‐3/NIR‐light dual activation of Pt(IV) prodrug **37**. Reproduced with permission: Copyright 2014, WILEY‐VCH.^[51]^ (b) Light activation of Pt(IV) prodrug **38**. Reproduced with permission: Copyright 2015, American Chemical Society.^[52]^

A NIR‐light‐responsive nanosystem was constructed through combining photo‐activable Pt(IV) prodrug and caspase‐3 imaging probes into core‐shell upconversion nanoparticles (UCNPs)@SiO_2_ for the remotely controlled activation of Pt(IV) prodrug **37** and real‐time monitor of apoptosis (Figure 16a).^[51]^ This NIR‐light‐activable Pt(IV) complex **37** flanked with one carboxyl moiety at the axial position with N‐hydroxysuccinimide (NHS) functionalization was selected to coordinate with the silica‐coated UPNPs. Simultaneously, a short peptide probe containing the NIR fluorescence donor Cy5 and quencher Qsy21, termed Cy5‐acp‐CGDEVDAK‐Qsy21, was linked on the UCNPs@SiO_2_ structure for specific recognition of caspase‐3 enzyme. As one of the essential executioner enzymes during the apoptotic process, the caspase‐3 can be activated by the fluorescent resonance energy transfer (FRET) process. When exposed upon 980 nm laser irradiation, active Pt(II) species is released from the Pt(IV)‐based UCNPs@SiO_2_ and the photocytotoxicity against both A2780 and A2780cis cells greatly enhances. It is worth noting that the obvious red fluorescence emitted by Cy5 can be detected in both A2780 and A2780cis cell lines after incubation with Pt(IV)‐based UCNPs@SiO_2_ through confocal laser scanning microscopy. The cleavage of the peptide probe by caspase‐3 enzyme was attributed to the disruption of the FRET process by releasing the Qsy21 away from the Cy5. The activated caspase‐3 along with the apoptotic cancer cells can be clearly observed in the confocal images.

As shown in Figure 16b, a similar photo‐activable Pt(IV) prodrug **38** was developed with a nanocarrier system by the conjugation with an HSA protein carrier.^[52]^ Sufficient amino groups were distributed on the HSA protein surface, thereby greatly improving the payload capacity of the nanocarrier system for Pt(IV) prodrug **38**. At the same time, the photo‐activated drug release induces higher cytotoxicity, resulting in cellular apoptosis and triggering the activation of caspase‐3. Caspase‐3, an essential protease enzyme during apoptosis, leads to the breakdown of the activable peptide unit (DEVD) linked with a FRET pair consisting of Cy5 and Qsy21 on the nanocarrier surface. This nanocarrier system exhibited light/enzyme dual‐responsive feature and released the cytotoxic Pt(II) species exposed upon the ultraviolet‐light irradiation, leading to improved cell killing effect in vitro towards both A2780cis and A2780 cancer cell lines. Meanwhile, the HSA nanodelivery system can function as a highly efficient cargo to increase the distribution of Pt(IV) prodrugs in A2780 and A2780cis upon the ultraviolet light irradiation.

### 4.5 Enzyme/redox‐activable metallo‐prodrugs

**FIGURE 17:**
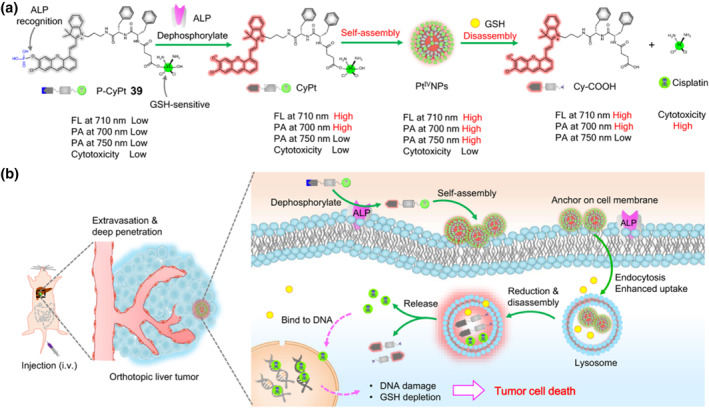
(a) Chemical structure of P‐CyPt **39**, which undergoes membrane‐bound ALP‐triggered dephosphorylation and in‐situ self‐assembly process into Pt(IV) nanoparticles, followed by GSH‐stimulated disassembly to rapidly release the fluorescent Cy‐COOH and cytotoxic cisplatin. (b) Mechanism of intravenous injection of P‐CyPt for in vivo fluorescent/photoacoustic bimodal imaging‐guided chemotherapy of HepG2 tumors. Reproduced with permission: Copyright 2023, Springer Nature.^[53]^

Recently, Wen et al. reported a sequential‐stimuli activable prodrug for the controlled delivery and drug release in vivo through an in situ self‐assembly and disassembly approach.^[53]^ In this study, they successfully developed an enzyme/redox dual‐trigger‐responsive cisplatin prodrug **39** (P‐CyPt) that would undergo membrane‐bound alkaline phosphatase (ALP)‐stimulated in‐situ self‐assembly, intracellular GSH‐stimulated reduction and disassembly process, thereby enhancing the local accumulation and eliciting the rapid release of fluorescent Cy‐COOH and cytotoxic cisplatin (Figure 17a). After systemic drug administration, P‐CyPt prodrug largely enhances the therapeutic efficacy while mitigates the off‐target side effects in tumor‐bearing nude mice with subcutaneous HeLa tumors and orthotopic HepG2 liver tumors, compared with that of solely cisplatin (Figure 17b). Additionally, P‐CyPt can be efficiently activated, and then produce strong NIR‐I fluorescence (at 710 nm) and NIR‐I photoacoustic imaging signals (at 700 and 750 nm), realizing good sensitivity, high spatial‐resolution, precise real‐time monitoring of drug delivery and release in vivo by the dual‐modality imaging at tumor sites. This smart approach integrates the edges provided by the in‐situ self‐assembly with those of intracellular disassembly, paving the way for the development of prodrugs with enhanced drug delivery and controlled release for cancer treatment.

### 4.6 Enzyme/hypoxia/redox‐activable metallo‐prodrugs

**FIGURE 18:**
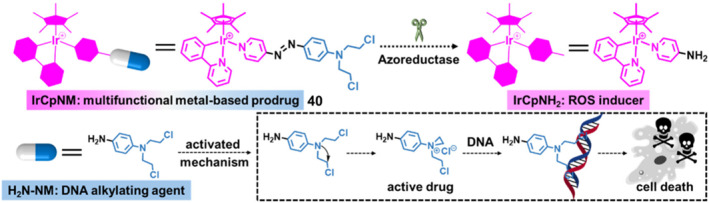
Design and activation of the azoreductase/hypoxia/redox‐responsive organo‐Ir(III) prodrug **40**, IrCpNM. Reproduced with permission: Copyright 2024, American Chemical Society.^[54]^

Recently, Wang et al. developed a smart enzyme/hypoxia/redox‐responsive organo‐Ir(III) prodrug **40**, termed IrCpNM, with the composition of the Ir‐arene subunit (a ROS‐inducing moiety), azo linker (a hypoxic‐stimuli moiety), and nitrogen mustard (N(CH_2_CH_2_Cl)_2_) subunit (a DNA‐alkylating moiety) (Figure 18).^[54]^ For the first time, the as‐synthesized IrCpNM prodrug achieved the DNA damage response‐mediated autophagy under hypoxia condition for lung cancer treatment. Azoreductase, an overexpressed enzyme in the hypoxic solid tumors, can efficiently reduce and cleave the azo groups. After the introduction of IrCpNM, the hypoxic condition was largely relieved, owing to the downregulation of hypoxia‐inducible factor‐1α (H1F‐1α) and the self‐generation of O_2_ from reduction of excessive H_2_O_2_ catalyzed by the elevated concentration of catalase. Next, the Ir‐arene subunit within IrCpNM facilitated the ROS production, and the cleavage of azo bonds by the elevated azoreductase and GSH resulted in the imbalance of redox homeostasis, leading to the severe oxidative stress on DNA damage. Moreover, the nitrogen mustard could react with DNA to generate a covalent linkage. The in vitro and in vivo experimental results confirmed that the quadruple synergistic activities of IrCpNM prodrug stimulated the DNA damage response‐mediated autophagy against hypoxic solid tumor, providing the insights for the design strategy of novel organometallic prodrug with tumor microenvironment‐responsive system.

### 4.7 Enzyme/pH/radiation‐activable metallo‐prodrugs

**FIGURE 19:**
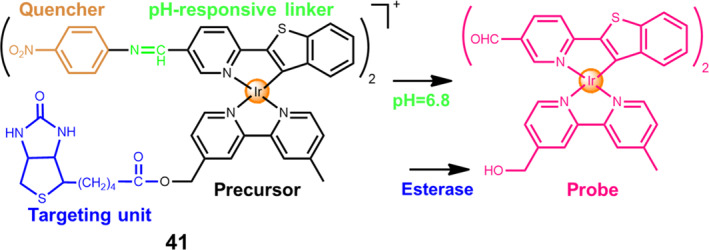
Chemical structure of esterase/pH/radiation multi‐stimuli‐activable Ir(III)‐based prodrug **41**, Ir‐NB.

Recently, Chen's group designed and constructed an enzyme/pH/radiation multi‐stimuli‐activable Ir(III)‐based prodrug **41**, namely Ir‐NB.^[55]^ Since it is highly sensitive to X‐rays, this novel tumor‐targeting Ir‐NB can function as a potential radiosensitizer for achieving bioimaging‐guided cancer radiochemotherapy. As shown in Figure 19, a tumor targeting moiety is linked with the radiosensitizer through an ester bond. Meanwhile, the cleavage of the acid‐sensitive imine bond can undergo breakdown in the weakly acidic environment and convert the Ir‐NB prodrug to the probe, thereby causing the enhancement and the red shifting in the emission spectra of the activated Ir(III) probe. The pH‐responsive linker is conjugated to a strong electron acceptor, a nitrobenzoyl moiety, which can quench the luminescence emission of the Ir(III) complex due to the photo‐induced electron transfer process. The experimental results prove that esterase enzyme and acidity synergistically contribute to the cleavage of the ester bond and the decomposition of the conjugates. Under the X‐ray radiation, this activated Ir(III) probe can achieve precise visualization of the tumor tissues within the xenograft nude mice. In addition to the X‐ray imaging, this Ir(III) complex containing high atomic number shows superior chemotherapeutic and radio‐sensitization potential towards A549 cancer cells due to the mitochondrial dysfunction triggered by oxidative stress, eventually facilitating enhanced apoptosis of A549 cells. This work provides an efficient strategy for the development of novel cancer radiochemotherapy agents.

## 5 CONCLUSION AND OUTLOOK

Stimuli‐activable metallo‐prodrugs are one of the most effective strategies to address the existing issues of metallodrugs, including severe toxicity, poor biodistribution, low aqueous solubility, and unfavorable reactions with off‐target biomolecules. Abundant stimuli‐activable metallo‐prodrugs have been reported utilizing endogenous‐ and/or exogenous‐stimuli as triggers, attributed to the complicated biological conditions. In this review, we enumerate the related paradigms of single‐, dual‐, or multi‐stimuli‐responsive metallo‐prodrugs with site‐specific delivery for cancer therapy in the past two decades. Table 1 is a summary of selected paradigms of stimuli‐responsive metallo‐prodrugs for cancer therapy.

**TABLE 1:** Selected examples of stimuli‐activable metallo‐prodrugs.

No.	Metal	Stimuli	Mechanism	Target	References
1	Pt	Low pH	DNA damage	DNA	[15a]
2	Cu	Low pH	DNA damage, mitochondrial dysfunction, p53‐activation, G2/M phase cell cycle arrest, apoptosis	Cytoplasm	[15b]
3	Ru	Low pH	DNA damage, apoptosis	Cytoplasm	[15c]
4	Pt	β‐lactamase	‐	‐	[16a]
5	Pt	β‐glucuronidase	‐	‐	[16b]
6	Fe	H_2_O_2_	DNA damage, ROS generation	Cytoplasm	[18a]
7	Ir	Fe^3+^	Hydrolyzed to ROS generator and Ir(III) complex, lysosomes damage, mitochondrial dysfunction, apoptosis, necroptosis	Lysosomes	[18b]
8	Pt	Cellular reductants	‐	DNA	[24]
9	Pt	Cellular reductants	DNA binding	DNA	[25]
10	Pt	Ascorbic acid	DNA binding, apoptosis	DNA	[26]
11	Pt	Cellular reductants	DNA binding, topoisomerase II inhibitor	DNA	[28]
12	Pt	Cellular reductants	DNA binding, DNA damage, apoptosis	DNA	[29]
13	Pt	Cellular reductants	Mitochondrial dysfunction, ROS generation, DNA damage, apoptosis	DNA	[30]
14	Au	GSH	Inhibition of the TrxR activity, ROS generation, DNA damage, ferroptosis	Cytoplasm	[31]
15	Ir	Ultraviolet light irradiation	Photocytotoxicity, apoptosis	Mitochondria	[32a]
16	Pt	Ultraviolet light irradiation	DNA platination, HDAC inhibition, RNA transcription blockage	DNA, HDAC	[32b]
17	Pt	Blue light irradiation	DNA damage, photocytotoxicity, p53‐activation, apoptosis, ICD	Nucleus	[33a]
18	Pt	Green light irradiation	DNA platination, photocytotoxicity, ROS generation, oncosis	DNA	[33b, 33c]
19	Pt	Red light irradiation	ROS generation, DNA binding, photocytotoxicity, apoptosis	DNA	[33d]
20	Ru	Green light irradiation	ROS generation, DNA binding, photocytotoxicity, apoptosis	DNA	[33e]
21	Ir	NIR light irradiation	Mitochondrial dysfunction, ROS generation, photocytotoxicity, apoptosis	Mitochondria	[34a]
22	Pt	NIR light irradiation	ROS generation, severe oxidative stress, upregulation of lipid oxidation metabolites, imbalanced pH homeostasis, photocytotoxicity, non‐classical necrosis, ICD	Endoplasmic reticulum, DNA	[34b]
23	Pt	NIR light irradiation	DNA damage, ROS generation, apoptosis	Nucleus	[34c]
24	Pt	Ultrasound	Mitochondrial DNA damage, ROS generation, depletion of cellular reductants (GSH and NADH)	Mitochondria	[35a]
25	Pt	Ultrasound	DNA damage, ROS generation, apoptosis	Nucleus	[35b]
26	Ru	X‐ray radiation	ROS generation, mitochondrial dysfunction by destroy the mitochondrial respiratory chain upon X‐ray radiation, apoptosis, ICD	Mitochondria	[36]
27	Pt	Visible light irradiation and GSH reduction	DNA binding, photocytotoxicity, ROS generation	DNA	[41]
28	Ru	Blue light irradiation, two‐photon NIR activation, GSH reduction	Photocytotoxicity, ROS generation, apoptosis	Mitochondria	[42]
29	Pt	NIR light irradiation and GSH reduction	ROS generation, GSH depletion, apoptosis, ICD	Cytoplasm	[43]
30	Ir	Two‐photon NIR activation, H_2_O_2_	GSH depletion, ROS generation, mitochondrial dysfunction, apoptosis	Mitochondria	[44]
31	Ru	Low pH and blue light irradiation	DNA damage, photocytotoxicity	DNA	[45]
32	Ru	Low pH and blue light irradiation	DNA binding, photocytotoxicity, apoptosis	DNA	[46]
33	Au	Low pH and red light irradiation	Photocytotoxicity, TrxR inhibition	Cytoplasm	[47]
34	Pt	Low pH and cellular reductants	DNA damage, apoptosis	DNA	[48]
35	Pt	Low pH and cellular reductants	Nuclear DNA platination and apoptosis	DNA	[49]
36	Pt	Low pH and GSH reduction	Mitochondrial Ca^2+^ overload, GSH depletion, ROS generation, nuclear DNA platination, apoptosis, ferroptosis, ICD	DNA, mitochondria	[50]
37	Pt	Caspase‐3 enzyme and NIR light irradiation	Photocytotoxicity, nuclear DNA platination, caspase‐3 activation, apoptosis	DNA	[51]
38	Pt	Caspase‐3 enzyme and ultraviolet light irradiation	Photocytotoxicity, nuclear DNA platination, caspase‐3 activation, apoptosis	DNA	[52]
39	Pt	Alkaline phosphatase enzyme and GSH reduction	DNA damage, GSH depletion	DNA	[53]
40	Ir	Azoreductase enzyme, hypoxia and H_2_O_2_/GSH reduction	DNA damage, mitochondrial dysfunction, GSH depletion, ROS generation, autophagy, upregulation of catalase, downregulation of HIF‐1α	Mitochondria, nucleus, and DNA	[54]
41	Ir	Esterase enzyme, low pH, and X‐ray radiation	DNA damage, mitochondrial dysfunction, apoptosis	Mitochondria	[55]

Although the utilization of single‐, dual‐, and multi‐stimuli to precisely deliver metallo‐drugs into malignant tumors displayed promising experimental results for cancer theranostics, some existing challenges need to be bridged. As far as we know, very few metallo‐prodrugs mentioned in this review have entered clinical trials. The future tasks of the metallo‐prodrug systems are to address the existing challenges by enhancing their targeting ability, precisely controlling the release of active species, improving the biodistribution in targeted tissues, and addressing the accumulation issues in favorable sub‐organelles. Another main issue is the limited systematic studies on the investigation of pharmacokinetics and evaluation of systematic toxicity in vivo. To date, there are few reports about the systematic investigations of biodistribution, absorption, metabolism and excretion behaviors in vivo. Additionally, some non‐classical therapeutic metallo‐drugs have displayed novel mechanisms of action which may function as smart stimuli‐activable metallo‐prodrugs. Despite that ligands show great impact on the activities of metallo‐prodrugs, central metal ions mainly decide their pharmacological effects. However, overwhelming investigations are focused on the metal complexes of transition metals, typically on those of platinum, ruthenium, iridium, and copper, while other metal complexes are often neglected nowadays. The overlooked metal complexes are probably endowed with some fascinating characteristics which could address the drawbacks of current stimuli‐activable metallo‐prodrugs. Finally, the exact behaviors and the specific mechanisms of actions in the physicochemical environment are not fully elucidated. We believe that the further in‐depth studies will facilitate in understanding the specific behaviors and exact mechanisms, thereby providing guidance for the rational design and practical application of stimuli‐responsive metallo‐prodrugs. In the near future, the stimuli‐activable metallo‐prodrugs hold substantial therapeutic potential as a complementary approach for the cancer theranostics.

## CONFLICT OF INTEREST STATEMENT

The authors declare no conflicts of interest.

## Data Availability

Data sharing not applicable—no new data generated.
